# Comparison of gut microbiota structure and *Actinobacteria* abundances in healthy young adults and elderly subjects: a pilot study

**DOI:** 10.1186/s12866-020-02068-z

**Published:** 2021-01-06

**Authors:** Jun Li, Haiyan Si, Haitao Du, Hongxia Guo, Huanqin Dai, Shiping Xu, Jun Wan

**Affiliations:** 1 Department of Gastroenterology, 2nd Medical Center, Chinese People’s Liberation Army General Hospital, No. 28 Fuxing Road, Beijing, 100853 China; 2 Department of Oncology, First Medical Center, Chinese People’s Liberation Army General Hospital, Beijing, China; 3 Chinese Academy Sciences Key Laboratory of Pathogenic Microbiology and Immunology, Institute of Microbiology, Chinese Academy of Sciences, Beijing, China

**Keywords:** Gut microbiota, *Actinomycetes*, KEGG metabolic pathways, Antibiotic resistant genes, Metagenomics

## Abstract

**Background:**

The aim was to determine the potential association of the gut microbiota composition, especially the abundance of *Actinobacteria*, as well as the differentiation of functional and resistance genes with age (young adults vs elderly subjects) in China.

**Results:**

The patterns of relative abundance of all bacteria isolated from fecal samples differed between young adults and elderly subjects, but the alpha diversity (Chao1 *P* = 0.370, Shannon *P* = 0.560 and Simpson *P* = 0.270) and beta diversity (ANOSIM R = 0.031, *P* = 0.226) were not significantly different. There were 3 Kyoto Encyclopedia of Genes and Genomes (KEGG) metabolic pathways (carbon metabolism, inositol phosphate metabolism, and sesquiterpenoid and triterpenoid biosynthesis) and 7 antibiotic resistant genes (ARGs) (macrolide lincosamide-streptogramin B (MLSB), tetracycline, aminoglycoside, sulfonamide, fosmidomycin, lincomycin, and vancomycin) that showed significant differences between the 2 groups (all *P* < 0.05). The abundance of *Actinomycetes* was enriched (about 2.4-fold) in young adults. Bifidobacteria dominated in both young adults and elderly subjects, with overall higher abundances in young adults (*P* > 0.05). Only the *Bifidobacterium_dentium* species showed significant differences between the 2 groups (*P* = 0.013), with a higher abundance in elderly subjects but absent in young adults.

**Conclusions:**

The present study revealed that there were 3 KEGG metabolic pathways and 7 ARGs as well as enhanced *Bifidobacterium_dentium* species abundance in elderly compared to young subjects.

**Supplementary Information:**

The online version contains supplementary material available at 10.1186/s12866-020-02068-z.

## Background

As the largest and most complex microbial community in the human body, the gut microbiota is also an important metabolic organ that participates in the metabolism of nutrients in the body [1]. It is also associated with the occurrence, development and outcomes of obesity, intestinal disease, liver disease, neuropsychiatric disease, tumors, cardiovascular disease, diabetes, and other ailments [2–8]. The gut microbiota is also thought to be a reservoir for antibiotic resistant genes, with close contact between bacteria resulting in the sustained and widespread proliferation of resistance [9]. Previous studies have shown that the host genotype, diet, antibiotics, age and various diseases all affect the composition and diversity of the gut microbiota [10–13]. The relationship between gut microbiota and human age remains controversial and few studies have compared gut microbiota diversity between healthy young adults and elderly subjects in China, mainly focusing on changes in microbial abundance. Although the composition of the gut microbiota is diverse and dynamic within a short time period, it remains remarkably stable among individuals and their family members over time [14].

However, due to enhanced exposure to antibiotic resistant genes in elderly subjects the resistance gene pattern in their microbiota becomes more complex than in young adults [15]. The intestine of a fetus is sterile, but a few hours after the birth of a newborn, microbiota begin to colonize the intestine rapidly, mainly consisting of facultative anaerobes such as *Streptococcus*, *Staphylococcus* and *Enterobacteriaceae.* However, approximately 4 days after birth, *Bifidobacterium* becomes the dominant microbiota in the gut [16, 17]. The composition of the gut microbiome in 3-year-old infants tends to be stable and is similar to that of healthy adults [18]. The gut microbiome of elderly subjects is less abundant, due to malnutrition, a weak intestinal barrier function, decreased gastric acid secretion and/or autoimmune activity, and is mainly reflected in a decrease in *Bacteroides*, *Lactobacillus* and *Bifidobacterium* species [19, 20]. *Ruminococcus*, which can decompose cellulose, is almost absent in the elderly, whereas it is dominant in healthy young adults [21]. A significant increase in putrefying bacteria including *Streptococcus*, *Enterobacteriaceae* and *Staphylococcus,* might easily lead to increased colonic spoilage and infectious diseases [22]. However, studies have shown that extremely old people seem to experience a parallel increase in health-associated species of *Akkermansia*, *Bifidobacterium* and *Christensenellaceae* [23].

With the rapid development of metagenomics and bioinformatics in recent years, next-generation sequencing technology, which effectively avoids shortcomings such as low throughput, low accuracy and complex operation, has been used to investigate microbial diversity and community characteristics, and has generated up to 1000 Mb of data [24]. *Actinomycetes*, which are Gram-positive prokaryotes with high guanine and cytosine (G + C, accounting for more than 70%), are widely found in nature and have a strong coding ability for secondary metabolites. Nearly half of all known bioactive compounds and 70% of antibiotics used in humans are derived from *Actinomycetes* [25]. In particular, *Bifidobacterium*, as one of the human intestinal symbiotic probiotics, is closely related to human health [26]. Actinobacteria taxonomy is an important subject in *Actinomycetes* research, and with the emergence of new strains, research is constantly evolving [27].

The present study is a preliminary investigation of the composition and diversity of gut microbiota in healthy young adults and elderly subjects in China. Based on next-generation high-throughput sequencing (HiSeq X10 platform), alpha diversity and abundance differences, changes in metabolic functions, and antibiotic resistant genes (ARGs) were evaluated. We also analyzed the taxonomy and abundance of gut Actinobacteria in healthy young adults and elderly subjects. Studying the characteristics of healthy subjects in different age groups not only helps us better understand the relationship between bacterial imbalance in different age groups, but also helps identify more suitable probiotics for the treatment of different age groups in clinical practice.

## Results

### Gut microbial abundances at different taxonomic levels in young adults and elderly subjects

Fecal samples were obtained from 8 healthy young adults and 8 healthy elderly subjects, with an average age of 33.3 ± 7.6 and 71.6 ± 5.8 years, respectively. Table 1 shows the demographic characteristics of the included subjects.

**Table 1:** Demographic characteristics of the included subjects

	Healthy young adults (***n*** = 8)	Healthy elder adults (***n*** = 8)	Total (***n*** = 16)
Age (years), mean ± SD	33.3 ± 7.6	71.6 ± 5.8	52.4 ± 20.9
Gender/male, n (%)	8 (100)	8 (100)	16 (100)
Smoker, n (%)	0	2 (25)	2 (12.5)
N-smoker, n (%)	8 (100)	6 (75)	14 (87.5)
BMI (kg/m^2^), mean ± SD	23.3 ± 2.4	25.9 ± 2.4	24.6 ± 2.7
Diet	N/A	N/A	N/A

After metagenomic sequencing analysis of 8 healthy young adults, there were 606.97 M high quality reads in total, and the average high-quality reads per individual was 75.82 M. Similarly, 389.24 M high quality reads (average 11.34 Gb per individual) were obtained from 8 healthy elderly subjects.

**Fig. 1:**
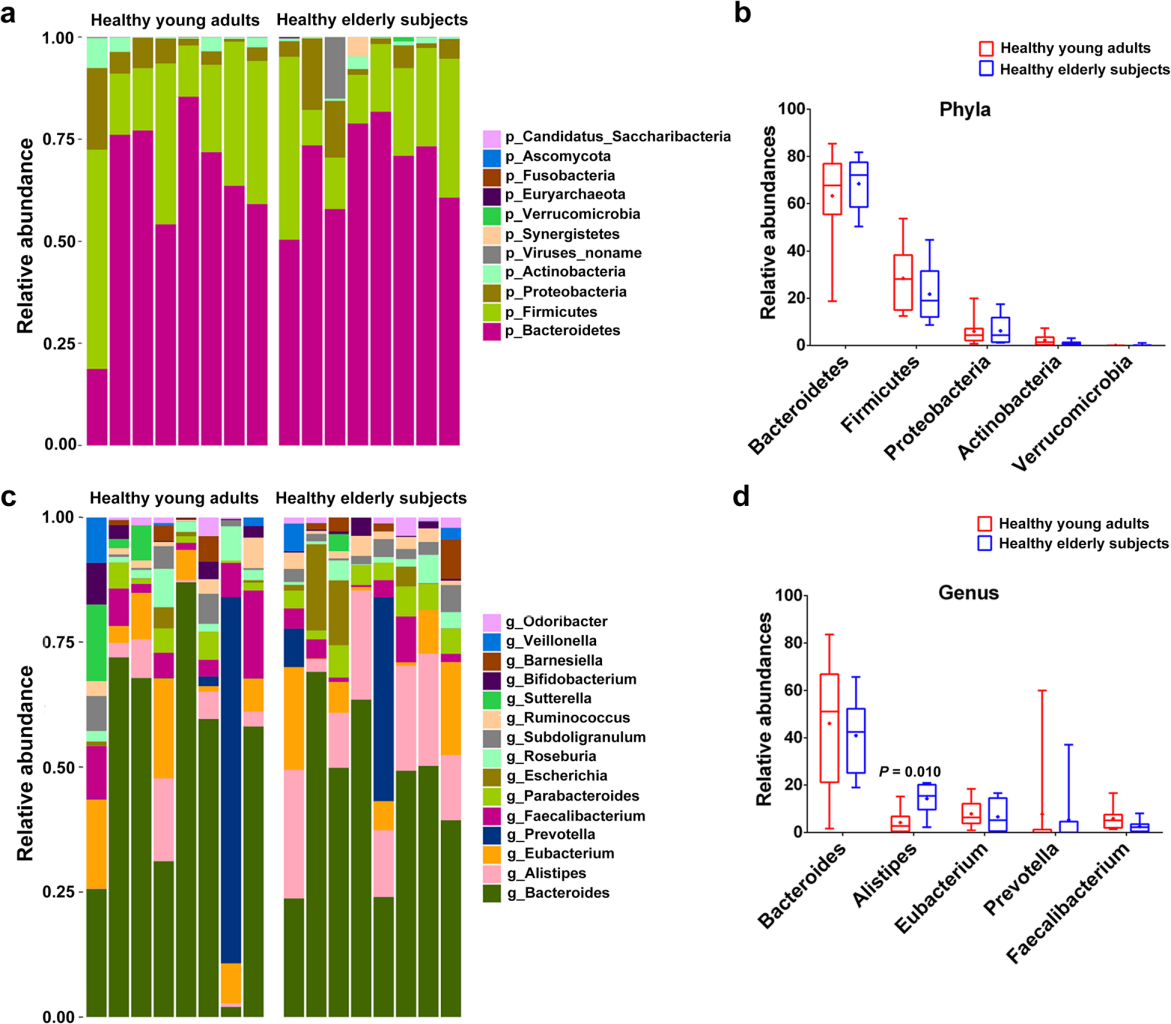
Relative abundances in gut microbiota at the levels of phylum and genus in healthy young adults and elderly subjects. (**a**, **c**) Bar-plot analysis shows the top 11 phylum abundances (or top 15 genus abundances) in each group; (**b**, **d**) Box-plot analysis shows the top 5 phylum or genus abundances in each group

In the all samples of young adults and elderly subjects, more than 99.0% strains of the gut microbiota were screened out at the level of kingdom, which were bacteria. Less than 1.0% were viruses, eukaryotes, which were found only in young adults, and *Archaea*, which were detected only in elderly subjects. Next, we compared the gut microbial composition at the phylum and genus levels between healthy young adults and elderly subjects. We found a total of 11 phylum abundances in each group, of which only *Synergistetes* was significantly enriched in elderly subjects (*P* = 0.013), but interestingly were not detected in young adults. The top 5 phyla were *Bacteroidetes, Firmicutes*, *Proteobacteria*, *Actinobacteria* and *Verrucomicrobia*. Compared with elderly subjects, only the relative abundance of *Firmicutes* and *Actinobacteria* in young adults seemed to be higher and the relative abundance of *Bacteroidetes, Proteobacteria,* and *Verrucomicrobia* appeared to be lower, but there statistical significance was not reached for these top 5 phyla (Fig. 1a). At the genus level, the top 5 gut microbiotas included *Bacteroides*, *Alistipes*, *Eubacterium*, *Prevotella* and *Faecalibacterium*. Among them, only *Alistipes* was enriched in elderly subjects, with a significant difference (*P* = 0.010; Fig. 1d). In addition, there were also differences in relative abundance between the 2 groups at the other levels (class, order, family and species; Additional file 1). Among the top 5 microbial abundances at the level of class, order, family and species in the 2 groups, *c_Betaproteobacteria*, *o_Burkholderiales* and s_Bacteroides_vulgatus abundance was significantly higher in healthy young adults (*P* < 0.05), whereas *f_Rikenellaceae* abundance was significantly higher in healthy elderly adults (*P* < 0.05).

### Gut microbiota diversity and composition differences between young adults and elderly subjects

**Fig. 2:**
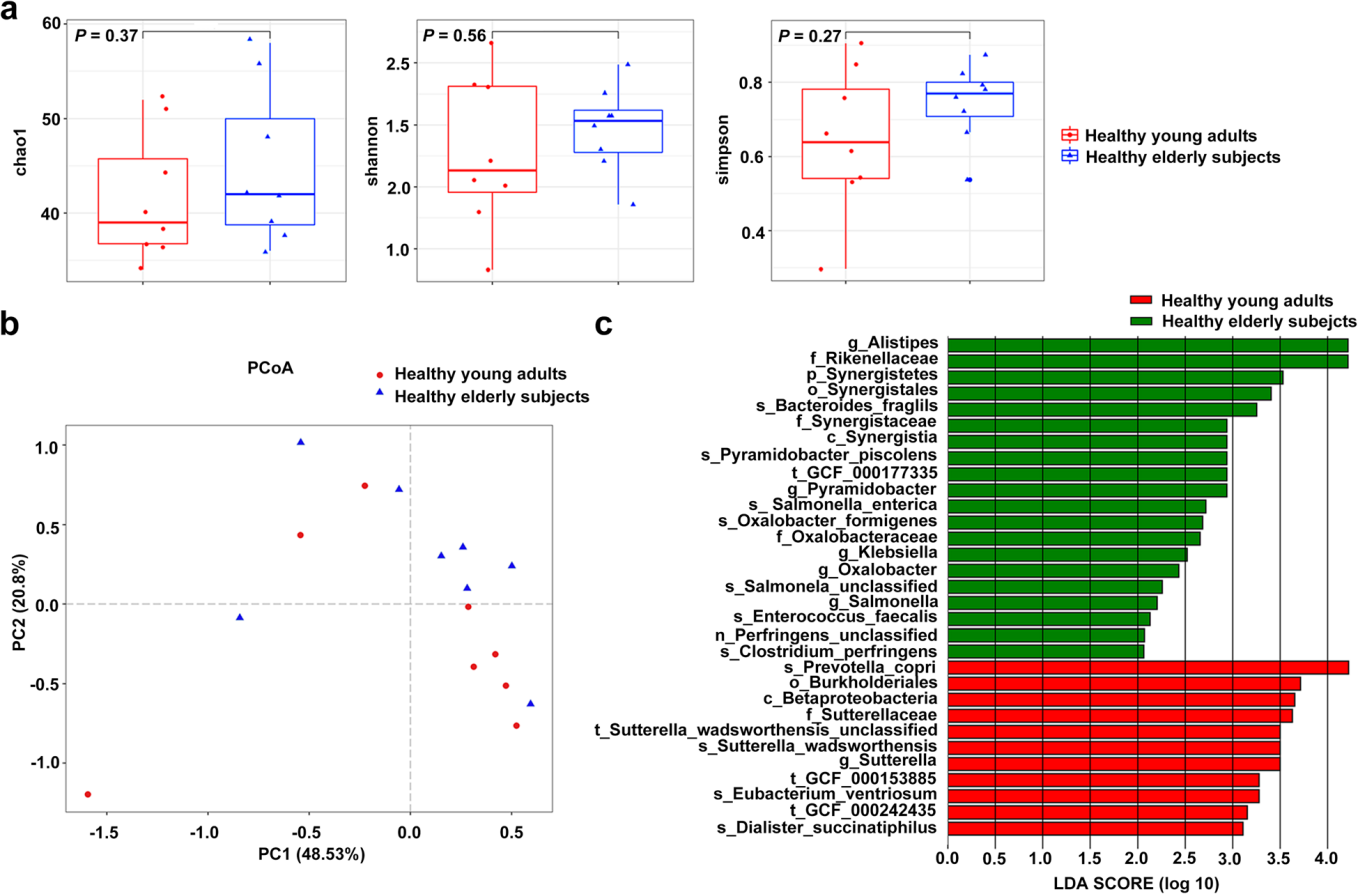
Comparison of α- and β-diversity at the genus level in gut microbiota between young adults and elderly subjects. **a** Comparison of α-diversity based on the Chao1, Shannon, and Simpson indices in each of the 2 groups; **b** Comparison of β diversity based on principal co-ordinates analysis (PCoA) in each of the 2 groups; **c** Distribution diagram of the LDA scores in each of the 2 groups and results of the LEfSe analysis based on the LDA scores to screen the species biomarkers (LDA > 2.0)

To explore alterations in the microbiota community structure between healthy young adults and elderly subjects, the Chao1, Shannon, and Simpson diversity indicators were used to estimate the alpha diversity at the level of the genus. As shown in Fig. 2a, the alpha diversity at the genus level appeared to be lower in young adults, but the apparent differences were not statistically significant (*P* = 0.370, *P* = 0.560, and *P* = 0.270, respectively). Similarly, the beta diversity analysis results revealed a total diversity difference between the 2 groups, and the contribution rate of the top 2 principal components was 62.33% (Fig. 2b), but the apparent difference in the microbiota composition was not statistically significant (ANOSIM R = 0.031, *P* = 0.226). To identify further the different biomarkers enriched in each group, LEfSe analysis was performed (Fig. 2c). Based on the LDA values, we found s_*Prevolla_copri*, *o_Burkholderiales*, *c_Betaproteobacter* and f*_Sutterellaceae* to be the dominant species in the young adults, and *g_Alistipes* and *f_Rikenellaceae* to be dominant in elderly subjects, with high LDA values (all LDA > 3.6). These results were consistent with the abundance results in s_*Prevolla_copri*, f*_Rikenellaceae*, *g_Alistipes*, *o_Burkholderiales* and *c_Betaproteobacter*.

### Comparison of gut microbiota metabolic functions in young adults and elderly subjects

**Fig. 3:**
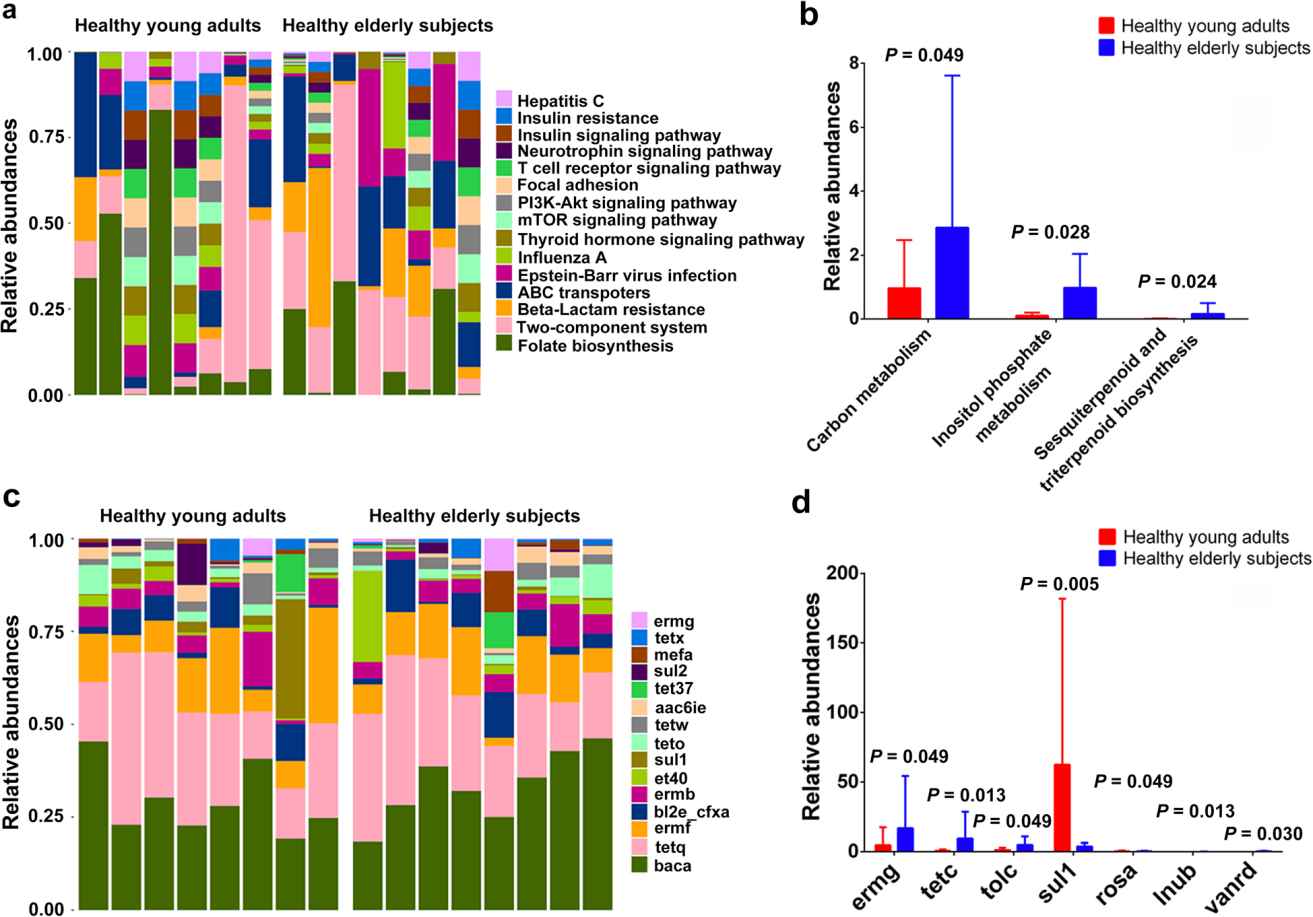
Relative abundances of KEGG metabolic pathway and antibiotic resistance gene (ARG) types in healthy young adults and elderly subjects. (**a**, **c**) Bar-plot analysis shows the top 15 abundances of the KEGG metabolic pathway/ARG types in each group; (**b**, **d**) Box-plot analysis shows the significant differences in KEGG metabolic pathway/ARG types in each group

To investigate the potential differences in metabolism between the 2 groups, we aligned the metagenomic data with the KEGG pathway and a total of 217 metabolic pathways were annotated. We found that only 3 metabolic pathways (carbon metabolism, inositol phosphate metabolism, and sesquiterpenoid and triterpenoid biosynthesis) showed significant differences between the 2 groups (all *P* < 0.05). As shown in Fig. 3c, d, the top 5 KEGG metabolic pathways in both groups were folate biosynthesis, Epstein-Barr virus infection, ABC transporters, histidine protein kinases system (two-component system), and beta-Lactam resistance. These results revealed that elderly subjects were all enriched with bacterial enzymes involved in carbon metabolism, inositol phosphate metabolism, and sesquiterpenoid and triterpenoid biosynthesis.

### Antibiotic resistant genes in the gut microbiota of young adults and elderly subjects

Recent studies have reported that the diversity of ARGs in human intestinal flora was age-related. Therefore, considering the differences in gut microbiota and metabolic pathways between young adults and elderly subjects revealed in this study, we further investigated the differences in ARGs between the 2 groups. A total of 101 ARG types (95 types in young adults and 98 types in elderly subjects) were obtained and annotated according to ARDB, of which, 7 ARG types (ermg, tetc, tolc, sul1, rosa, lnub, and vanrd) with significant differences were screened. The ARGs included macrolide lincosamide-streptogramin B (MLSB), tetracycline, aminoglycoside, sulfonamide, fosmidomycin, lincomycin, and vancomycin (all *P* < 0.05). Except for the higher levels of fosmidomycin in young adults, all others showed higher levels in elderly subjects. Lincomycin (lnub) was completely absent in young adults. Incidentally, the ARGs with the highest levels were bacitracin (baca), tetracycline (tetq), MLSB (ermf, ermb), cephalosporin (bl2e_cfxa) in both groups (Fig. 3). These results indicated that the ARGs in gut microbiota are different in young adults and elderly subjects, and the abundances likely increase with age.

### Differences in Actinobacteria composition between young adults and elderly subjects at various taxonomic levels

**Fig. 4:**
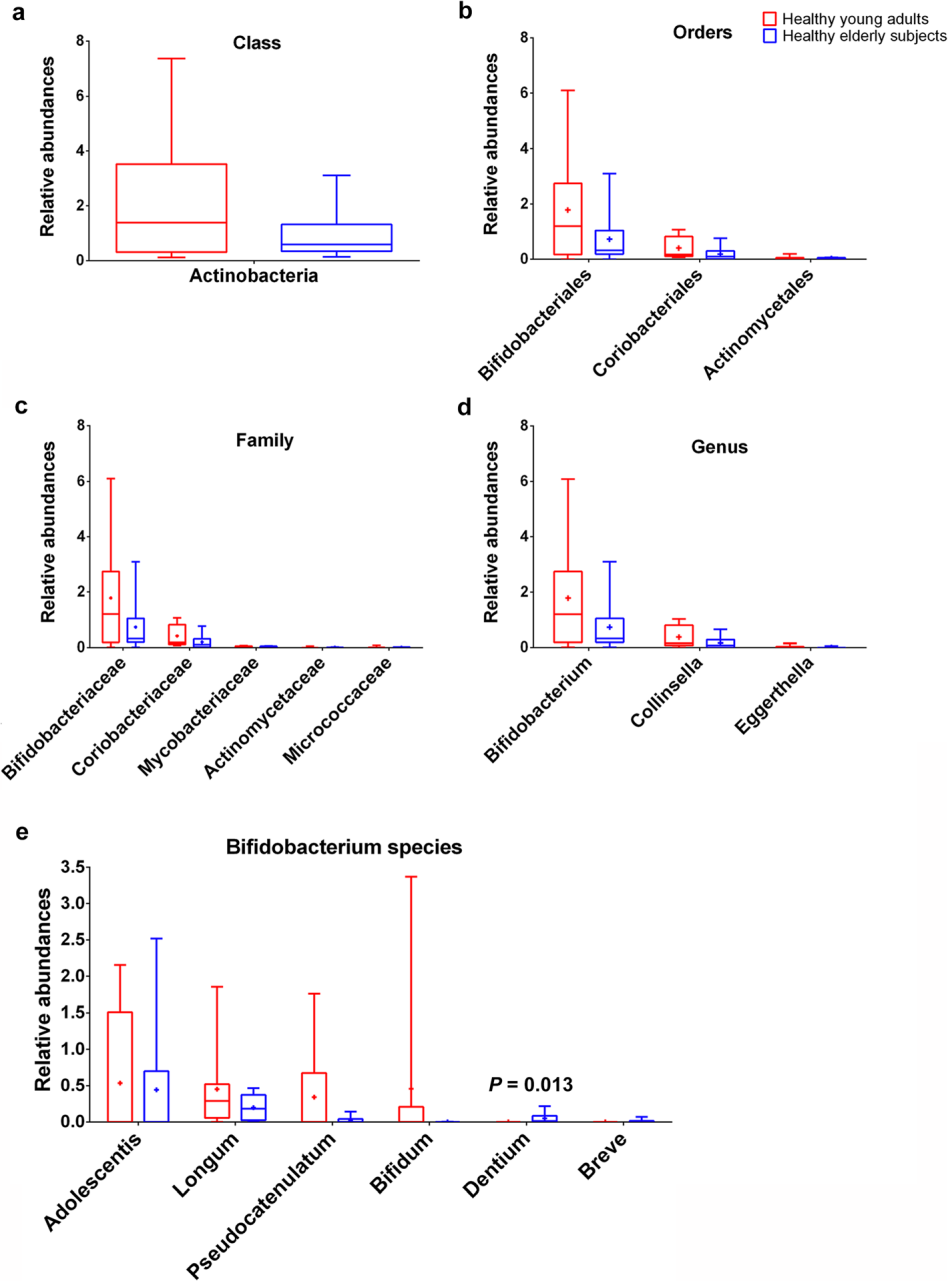
Relative abundances of *Acfinobacteria* at the levels of (**a**) class, (**b**) order, (**c**) family; (**d**) top 3 abundances of genus, and (**e**) Bifidobacterium species

As shown in Fig. 1 and Additional file 1, the relative abundances of *Actinomycetes* at the phylum and class levels were both apparently enriched (about 2.4-fold) in young adults, but without statistically significant difference. To analyze the differences in Actinobacteria composition between the 2 groups, the relative abundances at the levels of order, family, genus, and species were determined. At the level of orders, *Actinomycetales*, *Coriobacteriales*, and *Bifidobacteriales* were detected, and the data revealed that the relative abundances of these 3 genera both declined in elderly subjects. A total of 5 genera (*Bifidobacteriaceae*, *Coriobacteriaceae*, *Mycobacteriaceae*, *Actinomycetaceae* and *Micrococcaceae)* at the level of family, and 12 genera (*Bifidobacterium*, *Collinsella*, *Eggerthella*, *Rothia*, *Mycobacterium*, *Actinomyces*, *Gordonibacter*, *Atopobium*, *Adlercreutzia*, *Slackia*, *Alloscardovia*, *Olsenella*) at the level of genus were found between the 2 groups. Among them, the *f_Bifidobacteriaceae* and *g_Bifidobacterium* were the most dominant in the 2 groups and they were both lower in elderly subjects compared to young adults (*P* = 0.721). At the level of species, a total of 21 genera were detected that had some differences between young adults and elderly subjects, but only *B. Dentium* was significantly different between the 2 groups (*P* = 0.013), with a high abundance in elderly subjects and being absent in young adults. We also determined the relative abundances of other species that differed between the 2 groups regarding *Bifidobacterium;* however, the differences were not significant. As shown in Fig. 4, *B. Pseudocatenulatum*, *B. Bifidum*, *B. Longum*, and *B. Adolescentis* were all enriched in young adults, while *B. breve* had a higher abundance in elderly subjects. Among the *Bifidobacterium* species, *B. Adolescentis* was found to be dominant in both groups, and no *B. Bifidum* was detected in elderly subjects, while no *B. breve* was detected in young adults. These results indicated that the overall abundances of gut Actinobacteria, especially *Bifidobacteria,* gradually showed a decreasing trend with age, but there were still some elderly subjects in whom *Bifidobacterium* was enriched.

## Discussion

To the best of our knowledge, this pilot study is the first to compare gut microbiota composition, metabolic functions and ARGs between young adults and elderly subjects in China by using next-generation high-throughput sequencing (HiSeq X10). We also analyzed Actinobacteria composition and abundances at different taxonomic levels in these 2 groups.

Similar to previous studies [28, 29], we found that *Bacteroidetes*, *Firmicutes* and *Proteobacteria* were the predominant phyla in healthy subjects, and that *Firmicutes*, *Bacteroidetes,* and *Proteobacteria* were enriched in elderly subjects compared to young adults (*P* > 0.05). Results of previous studies that investigated microbial abundance at the genus level have been somewhat varied. Biagi et al. highlighted the presence of a core microbiota (*Bacteroides*, *Roseburia*, *Lachnospira* and *Dialister*) in healthy individuals aged 22 to 109 years, in which *Bacteroides* was the most abundant in the gut microbiota, but the abundance varied little with age [23]. Jeffery et al. reported that the abundances of core microbiota such as *Bacteroides*, *Alistipes* and *Parabacteroides* were greater in elderly subjects, with the most diverse microbiota being *Coprococcus*, *Prevotella* and *Catenibacterium*, which were easily affected by diet [30]. In the present study, we found that the abundance of *Alistipes* was significantly greater in elderly subjects (*P* < 0.05), while the abundances of *Bacteroides* and *Prevotella* were not significantly different.

Alpha and beta diversities were not significantly different between young adults and elderly subjects, but LDA analysis showed s_*Prevolla_copri*, *o_Burkholderiales*, *c_Betaproteobacter* and *f_Sutterellaceae* to be dominant in young adults, and *g_Alistipes* and *f_Rikenellaceae* to be dominant in elderly subjects (LDA > 3.6). These results indicated that the dominant microbiota in young adults decreased with age, along with increased abundance of other gut microbiota, thus forming new dominant microbiota in elderly subjects.

The abundance of *Actinomycetes* declined (about 2.4-fold) in elderly subjects, in whom the dominant microbiota *f_Bifidobacteriaceae* and *g_Bifidobacterium* had a tendency to decrease compared with young adults, but without significant difference (*P* = 0.721). These results were consistent with a previous study, which noted that the abundance of *Bifidobacterium breve* and *Bifidobacterium dentium* in fecal microbiotas of young adults were similar to elderly subjects (*P* > 0.05), but there was a trend of difference between young adults and elderly subjects, which needs a larger sample scale to be confirmed [13]. The reason why some elderly adults maintained high *f_Bifidobacteriaceae* and *g_Bifidobacterium* levels in their microbiota might be due to diet [31] or individual factors [32].

Next, we focused on the abundances of species in which *Bifidobacterium* dominated, and a total of 7 species were identified, but only *B. Dentium* differed significantly between the 2 groups (*P* = 0.013), with a high abundance in elderly subjects and being absent in young adults. In fact, *B. Dentium,* which is considered to be an opportunistic pathogen, was found to be associated with the development of oral caries (mainly dental caries) [33]. Genome analysis also revealed that *B. Dentium* had extensive genetic capabilities to metabolize a much larger variety of carbohydrates [33]. This finding might be related to the significant increase in carbon metabolism and inositol phosphate metabolism pathways in elderly subjects identified in the present study. Among *Bifidobacterium* species, *B. Adolescentis,* which dominated in both groups, was enriched in young adults. This finding is consistent with previously published reports, according to which the *B. Adolescentis* taxon exhibits greater genetic diversity, and can be found where plant polysaccharides are present in high abundance, such as the human large intestine [34].

The long-term use of antibiotics in clinical practice disrupts the dynamic balance of gut microbiota, increasing the number of pathogens, including opportunistic pathogens, in the gut microbiota [35, 36]. Because of the greater number of antibiotics and higher frequency of antibiotic use in China, there are more drug-resistant gene subtypes in the intestine of the Chinese population than in those living in other countries [37]. DNA microarray analysis has revealed an increase in ARGs with age [15], and the increased abundances in MLSB, tetracycline, aminoglycoside, sulfonamide, lincomycin and vancomycin in elderly subjects in this study may be attributable to long exposure to environment, food, drinking water and antibiotic treatment among other factors [37–40]. Sulfonamides are commonly used as feed additives in animal husbandry, and high levels of fosmidomycin in young adults may be due to the far greater consumption of meat in their diets compared to elderly subjects [41]. *Bifidobacterium* is frequently used as a probiotic supplement, because *Bifidobacterium* with moderate ARGs, combined with antibiotics, can inhibit the growth of pathogenic bacteria and regulate the gut microbiota, which is beneficial to the homeostasis of the host intestine. Hence, it is necessary to prevent the horizontal gene transfer (HGT) of ARGs in the *Bifidobacterium* employed [42].

This pilot study had a number of limitations: First, the microbiota results were obtained using a small sample size, and need to be confirmed by studying larger cohorts of subjects and the study is limited to functional and taxonomic mapping alone without including Metagenome Assembled Genomes (MAGs).

## Conclusions

We analyzed the abundances, diversity, metabolic pathway and ARGs of gut microbiota, and confirmed the composition of *Actinomycetes* in healthy young adults and elderly subjects. We found a significant increase of *B. Dentium* abundance in elderly subjects, which might be related to the metabolic pathways. Our study indicated that the alterations in gut microbiota composition and in functional and resistant genes may be related to age. Further studies are warranted to determine the potential mechanisms involved in these age-related differences.

## Methods

### Subjects

Healthy subjects were recruited from the outpatient department of the 8th Medical Center of Chinese People’s Liberation Army General Hospital between November 10, 2017 and December 15, 2018. Inclusion criteria for both healthy young adults (aged 18 to 45 years) and elderly (over 60 years of age) subjects were: male gender; in good health; without active gastrointestinal inflammation or bleeding; inflammatory bowel disease (IBD); tumors; diabetes mellitus; a previous history of major gastrointestinal surgery; and without special feeding history (e.g., vegan). Subjects were excluded if: they had received antibiotics, antifungal drugs or probiotics within 2 months prior to the start of the study; had diarrhea or constipation within 2 weeks prior to the start of the study; healthy elderly subjects who had a < 3 months active phase of chronic diseases such as hypertension and coronary heart disease.

Our study was performed in accordance with the principles of the Declaration of Helsinki with regard to ethical research involving human subjects, and the protocols were approved by Medical Ethics Committee of Chinese People’s Liberation Army General Hospital (approval No. S2018–081-02).

### Fecal sample collection processing

Each subject was pre-padded with a sterile urine pad and, after defecation, the central part of the feces was removed from the pad with a sterile spoon and placed in a sterile box. The sample in the box was transferred for preservation into a freezer at − 80 °C until DNA extraction. The time between fecal sampling and its placement in the − 80 °C freezer was < 3 h.

### DNA extraction and metagenomics sequencing

Total genomic DNA was extracted from 250 mg fecal samples using the QIAamp PowerFecal DNA kit (QIAGEN, Dusseldorf, German), according to the manufacturer’s instructions. The concentration and purity of genomic DNA was measured by D260/280 with Nanodrop spectrophotometer (Thermo Fisher Scientific, Waltham, MA, US) and 1% agaric gel electrophoresis (100 V, 60 min, with λ-Hind marker III). DNA was fragmented to an average size of approximately 450 bp by using NexteraTM DNA Sample Pre Kit (Illumina Inc., San Diego, CA, USA) and for paired-end library construction. The size distribution of the resulting fragments selected for amplification ranged on average from 500 to 700 bp. Subsequently, metagenomic sequencing was performed on a HiSeq X10 platform (Illumina Inc., San Diego, CA, USA), according to the manufacturer’s protocols. After sequencing, all sequence reads were pre-processed to remove low quality or artefactual bases. FastQC ver. 0.11.8 was used to assess the quality of the raw data, and Trimmomatic ver. 0.39 was used to trim the raw sequence reads. Reads were de novo assembled using MEGAHIT ver. 1.2.7 [43].

### Bioinformatics and statistical analysis

The output from Trimmomatic was used for taxonomic profiles and species annotations which were generated using MetaPhlAn2 with the internal marker gene set. MOCAT2 was used for functional annotation, including KEGG and ARGD [44], the results of which translated to relative abundance at the level of kingdom, phylum, class, order, family and species. Differences in alpha diversity were calculated by the Chao1, Shannon, and Simpson diversity indices, and further principal co-ordinates analysis (PCoA) and analysis of similarities (ANOSIM) were also performed. Briefly, stratified abundances of metagenomic functions were first renormalized after excluding any “unclassified” relative abundance. Contributional diversity for a given metagenomic function was then calculated by applying ecological similarity measures to the stratified abundance of that function. Gini-Simpson index was used for alpha-diversity and Bray-Curtis dissimilarity was used for beta-diversity. Statistically significant effects of biomarkers in the relative abundance of genera were performed using linear discriminant analysis (LDA) effect size (LEfSe). Only LDA values > 2.5 at a *P* value < 0.05 were considered significantly enriched. Functional annotation and abundance analysis were evaluated with Kyoto Encyclopedia of Genes and Genomes (KEGG). Antibiotic Resistance Genes Database (ARGD) was used to identify the ARGs, antibiotic resistance class, and corresponding types of antibiotics. Statistical analysis was performed with R-software (ver. 3.3.2) to calculate the alpha diversity metrics and GraphPad Prism ver. 6 (IBM, Armonk, NY, US). Significant difference between 2 groups was evaluated using a Wilcoxon rank sum test, with the level of significance being set at *P* < 0.05.

## Supplementary Information

**Additional file 1.** The top 5 abundances of all bacteria isolated from fecal samples of healthy young adults and elderly subjects at the level of (a) class, (b) order, (c) family, and (d) species.

## Data Availability

The datasets used and/or analyzed during the current study are available from the corresponding author on reasonable request.

## Abbreviations

ANOSIM: Analysis of similarities
ARGD: Antibiotic Resistance Genes Database
ARGs: Antibiotic resistance genes
HGT: Horizontal gene transfer
IGC: Integrated gene catalog
KEGG: Kyoto Encyclopedia of Genes and Genomes
LDA: Linear discriminant analysis
MLSB: Macrolide lincosamide-streptogramin B
PCoA: Principal co-ordinates analysis
