# Supplementary figures and images for: Comparison of gut microbiota structure and Actinobacteria abundances in healthy young adults and elderly subjects: a pilot study
Source: BMC Microbiol. 2021 Jan 6;21:13. doi: 10.1186/s12866-020-02068-z (PMC7788997; doi:10.1186/s12866-020-02068-z)

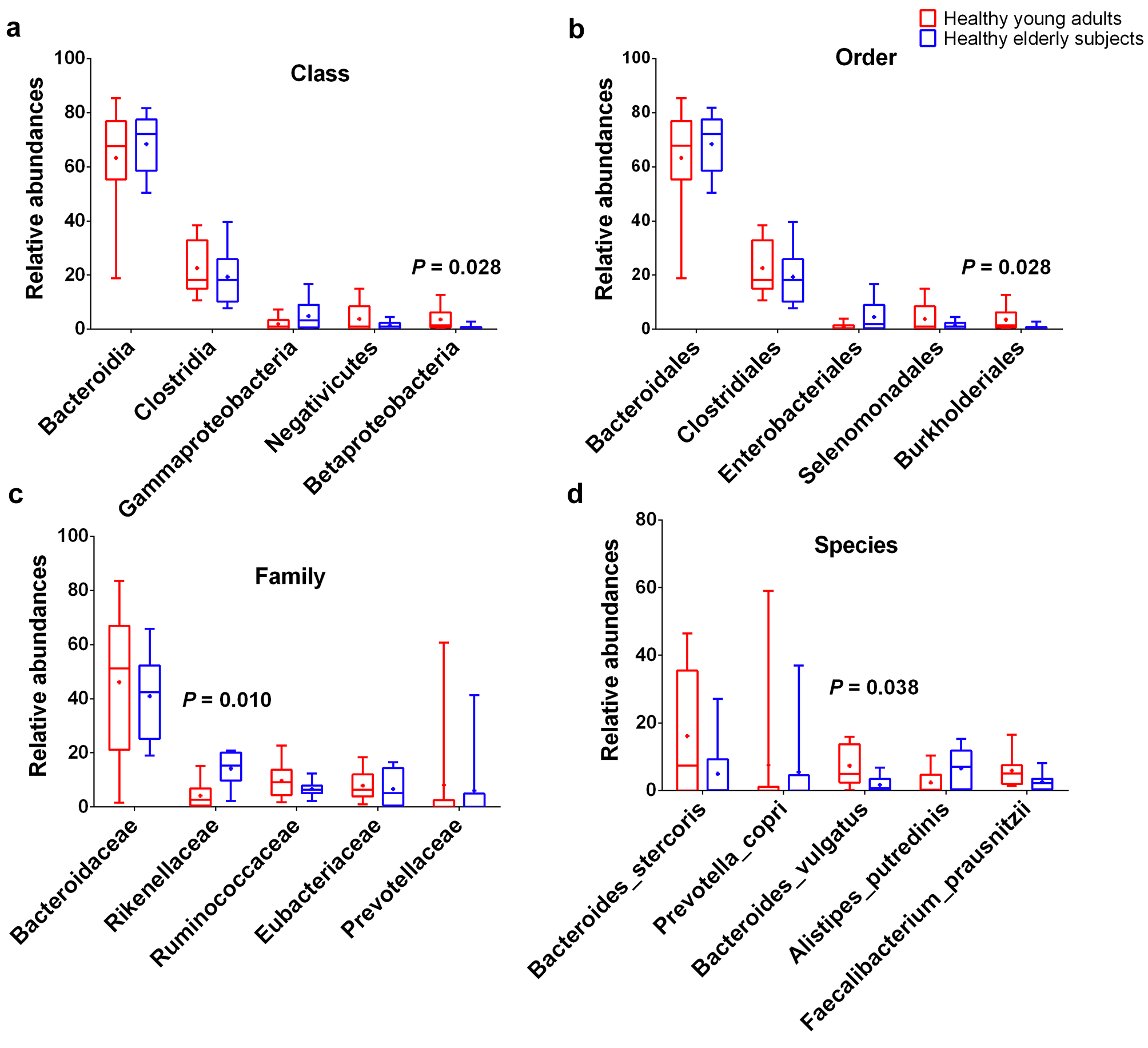

Supplement: Supplementary file 1 — Additional file 1. The top 5 abundances of all bacteria isolated from fecal samples of healthy young adults and elderly subjects at the level of (a) class, (b) order, (c) family, and (d) species. [file 12866_2020_2068_MOESM1_ESM.tif]
